# Safety and efficacy of peroral endoscopic myotomy with endoscopic fundoplication compared with POEM alone: International multicenter cohort study

**DOI:** 10.1055/a-2655-6550

**Published:** 2025-08-07

**Authors:** Michel Kahaleh, Vera Hapshy, Juan A Alcívar, Jorge Baquerizo-Burgos, Hannah Lukashok, Monica R Gaidhane, Iman Andalib, Amy Tyberg, Avik Sarkar, Haroon Shahid, Abid Allehibi, Resheed Alkhiari, Magda L Rodriguez, Carmen Bautista-Altamirano, Sarbelio Rodriguez, Maria G Porfilio, Mine Carames, Juan Carlos Carames, Amol Bapaye, Carlos Robles-Medranda

**Affiliations:** 1 Foundation of Interventional and Therapeutic Endoscopy, New York, United States; 2 23498 Gastroenterology, Hackensack Meridian Jersey Shore University Medical Center, Neptune City, United States; 3 Instituto Ecuatoriano de Enfermedades Digestivas, Guayaquil, Ecuador; 4 24263 Gastroenterology, Hackensack Meridian JFK University Medical Center, Edison, United States; 5 23498 Medicine, Hackensack Meridian Jersey Shore University Medical Center, Neptune City, United States; 6 37849 Gastroenterology, King Fahad Medical City, Riyadh, Saudi Arabia; 7 37850 Division of Gastroenterology, Department of Medicine, College of Medicine, King Saud University, Riyadh, Saudi Arabia; 8 537694 Gastroenterology, Our Lady of Remedies Clinic, Cali, Colombia; 9 Bariatric Endoscopy Unit, Hospital Universitario Ruber Juan Bravo, Madrid, Spain; 10 Endoscopy Unit, Service of Gastroenterology, Hospital 12 de Octubre, Madrid, Spain; 11 Gastroenterology, Escuela Dr.Ramon Madariaga, Posadas, Argentina; 12 Gastroenterology, Santander Hospital, Reynosa, Mexico; 13 Shivanand Desai Center for Digestive Disorders, Deenanath Mangeshkar Hospital and Research Center, Pune, India; 14 Endoscopy, Omni Hospital, Guayaquil, Ecuador

**Keywords:** Endoscopy Upper GI Tract, Motility / achalasia, POEM

## Abstract

**Background and study aims:**

Gastroesophageal reflux (GERD) can occur in a significant number of achalasia patients undergoing post-peroral endoscopic myotomy (POEM). POEM with endoscopic fundoplication (POEM-F) is a new endoscopic technique to treat post- POEM GERD. We conducted a multicenter cohort study to compare outcomes between POEM and POEM-F.

**Patients and methods:**

We included patients who underwent POEM or POEM-F from six tertiary centers. Primary outcomes were: 1) clinical success, defined as post-procedure cessation or reduction of proton-pump inhibitors; 2) post procedure percent time pH < 4 and DeMeester score; and 3) post-procedure Eckardt scores. Secondary outcomes included adverse events, procedure time, and hospital stay duration.

**Results:**

Sixty-four patients were included: 31 patients underwent POEM-F (mean age 51, 48% male), whereas 33 patients POEM (mean age 56, 58% male). Technical success was 100% in both groups. POEM-F patients achieved reduction/cessation in proton pump inhibitor use in 25 of 31 patients (80%); POEM patients in eight of 33 (24%) ( *P* ≤ 0.00001). Percent time with pH < 4 was significantly lower in the POEM-F group (2.75 +/- 2.53 vs 9.3 +/- 3.6 min, *P* ≤ 0.0001). Post-procedure DeMeester scores were lower (< 14.7) in POEM-F (mean 9.6) than POEM (mean 15.8) ( *P* ≤ 0.0011). There were three mucosal injuries in the POEM-F group and four in the POEM group. Hospital stay duration and procedure time did not differ between groups. In the POEM group, four patients required Dor fundoplication. No repeat interventions were recommended for the POEM- F group at 6-month follow-up.

**Conclusions:**

POEM-F achieves statistically significant improvement in post- POEM GERD compared with POEM alone.

## Introduction

Achalasia, an esophageal motility disorder characterized by impaired lower esophageal sphincter relaxation and absent peristalsis, significantly impacts patient quality of life 1 . Achalasia has been treated with a variety of methods, including pneumatic dilation, laparoscopic Heller myotomy, and more recently, peroral endoscopic myotomy (POEM) 2 . POEM, a minimally invasive endoscopic procedure, has emerged as a gold-standard treatment due to its high efficacy in relieving dysphagia and short-term recovery benefits 3 . However, post-POEM gastroesophageal reflux disease (GERD) remains a critical concern, with studies reporting incidence rates as high as 30% to 50%, necessitating long-term proton-pump inhibitor (PPI) therapy or additional interventions 4 . Absence of an anti-reflux mechanism during standard POEM contributes to this complication, prompting exploration of hybrid techniques such as POEM with concomitant endoscopic fundoplication (POEM-F). POEM-F aims to combine the myotomy of POEM with an endoscopic fundoplication at the gastroesophageal junction by wrapping the fundus of the stomach around the lower esophageal sphincter to help reduce incidence of GERD post-procedure 5 6 .

POEM-F represents a promising advancement in endoscopic management, combining myotomy with anti-reflux maneuvers in a single procedure. Early case series suggest that POEM-F may reduce post-procedure GERD by replicating surgical fundoplication principles endoscopically, but its broader applicability and long-term durability are understudied 6 . Long-term durability at 3-year follow-up has recently been reported in a single-center case-control study 7 Current literature lacks comparisons of objective GERD metrics—such as pH monitoring and DeMeester scores—between POEM-F and POEM, creating uncertainty about its role in clinical practice. This gap underscores the need for more studies to evaluate whether benefits of POEM-F translate into measurable improvements in GERD control without compromising procedure safety or efficiency.

This multicenter comparative study directly compared POEM-F and POEM in managing achalasia, with a focus on post-procedure GERD outcomes. We assessed clinical success (PPI cessation/reduction), objective reflux parameters (% time pH < 4, DeMeester score), and functional outcomes (GERD Health-Related Quality of Life (GERD-HRQL) and Eckardt scores) alongside adverse events (AEs) and procedure metrics. By collecting data from six tertiary centers, this study aimed to clarify whether POEM-F offers an advantage compared with POEM alone. The findings seek to inform evidence-based procedure selection, emphasizing patient-centered outcomes while addressing unresolved questions about the necessity of anti-reflux measures in POEM.

## Patients and methods

### Study population

We conducted a multicenter, retrospective, comparative study. Patients included met the following criteria: 1) age 18 years or older with diagnosis of achalasia; and 2) underwent either a POEM or a POEM-F at any of the participating tertiary care centers.

Overall, a total of 64 participants were included between May 2022 and May 2024 with a minimum follow-up time of 6 months (6–28 months). The study group (n = 31) included participants who underwent POEM-F and the control group (n = 33) included contemporary participants who underwent the POEM procedure. Patient demographics and medical history were collected and entered in a dedicated registry. Demographic data included age, gender, and body mass index. In addition, clinical data included pre- and post-procedure scoring systems validated for achalasia and GERD severity, which included GERD-HRQL, Eckardt symptom score, and DeMeester score. Other clinical characteristics included use of PPIs, history of esophagitis, and pH measurements. Procedure characteristics were also collected and analyzed. Follow-up visits at 1, 3, and 6 months were conducted with esophagogastroduodenoscopy and pHmetry and manometry studies at each of those intervals. All patients were started on PPIs post POEM to facilitate healing post POEM and titrated down based on symptoms.

All data were gathered in a registry (NCT05041608) approved by WCG IRB allowing the analysis of demographic, clinical, procedural, and follow-up data for all patients.

### Outcomes

Primary outcomes were assessed as following: 1) clinical success of procedure, defined as post-procedure cessation or reduction of PPIs; 2) post-procedure percent time pH < 4 as measured by 24-hour esophageal pH monitoring tests and post-procedure DeMeester score and GERD as well as absence of esophagitis; and 3) post-procedure Eckardt scores. Total post-procedure percent time pH < 4 and DeMeester score to quantify GERD were chosen because they are the two static parameters with the highest sensitivity and remain the gold standard in defining GERD 8 9 . Eckardt score was chosen to reliably define symptom severity of achalasia post-procedurally, as previously reported 10 . Secondary outcomes were evaluated, such as rates of AEs reliably attributed to the POEM procedure, total procedure time, and length of hospital stay.

### Procedure description

**Fig. 1:**
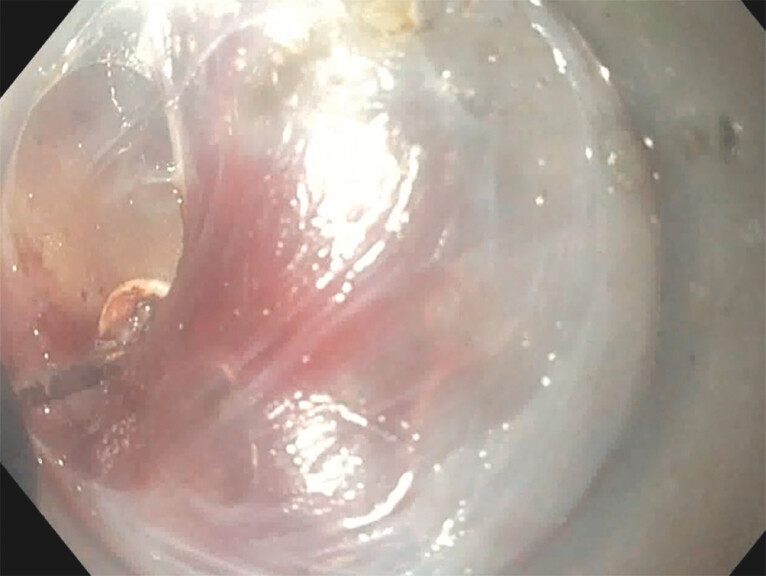
Opening of the gastric serosa.

**Fig. 2:**
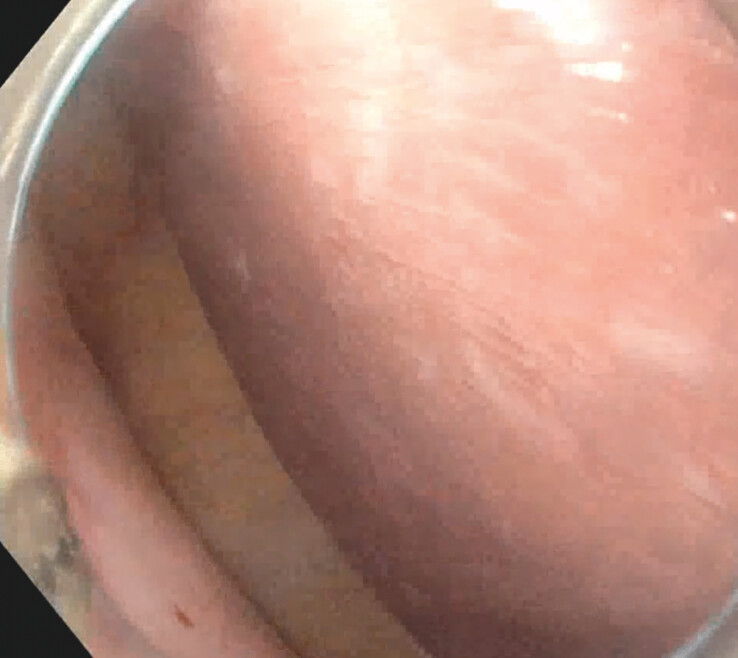
Entrance into the peritoneum with liver seen facing the stomach.

**Fig. 3:**
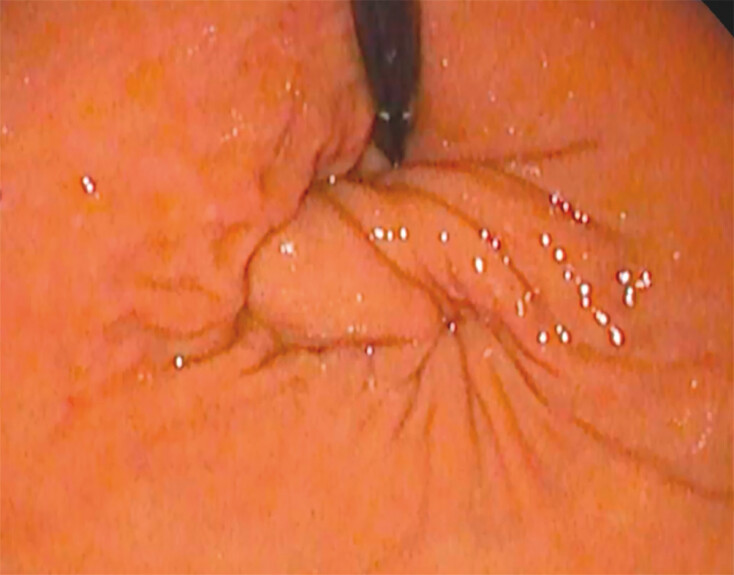
Retroflexion of the esogastric junction before fundoplication simulation.

**Fig. 4:**
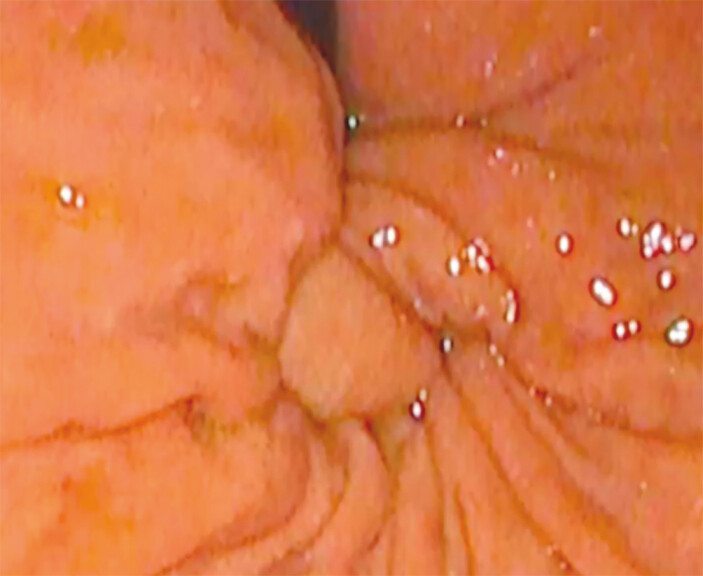
Retroflexion of the esogastric junction during fundoplication simulation.

**Fig. 5:**
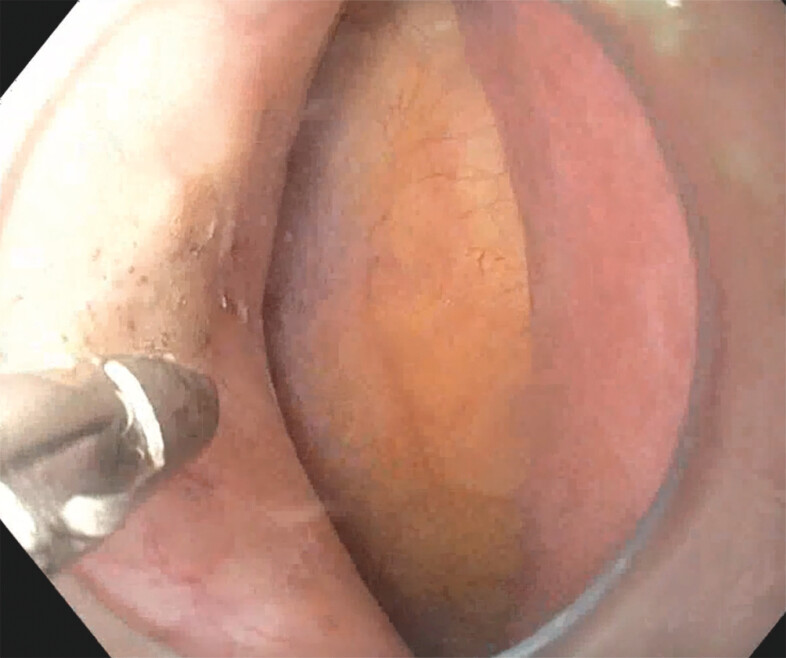
Marking of the stomach with diathermy.

**Fig. 6:**
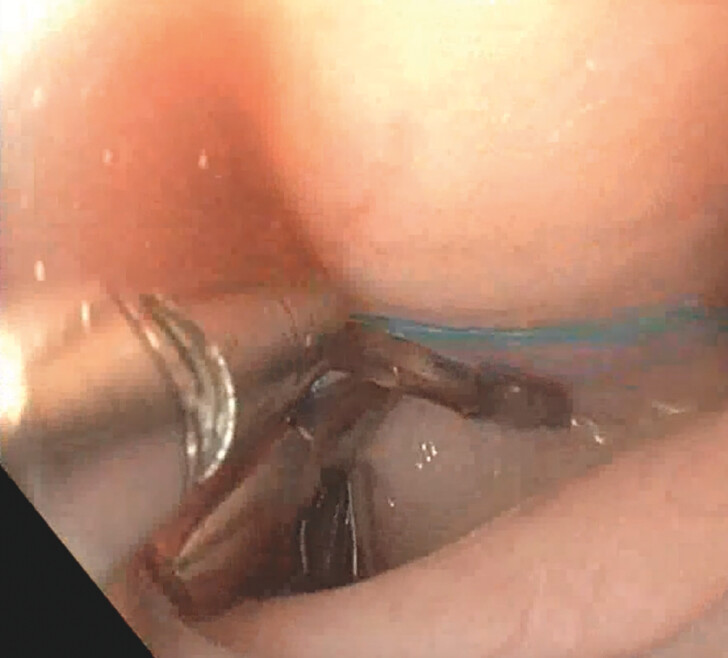
Clips over endoloop at the stomach level previously marked.

**Fig. 7:**
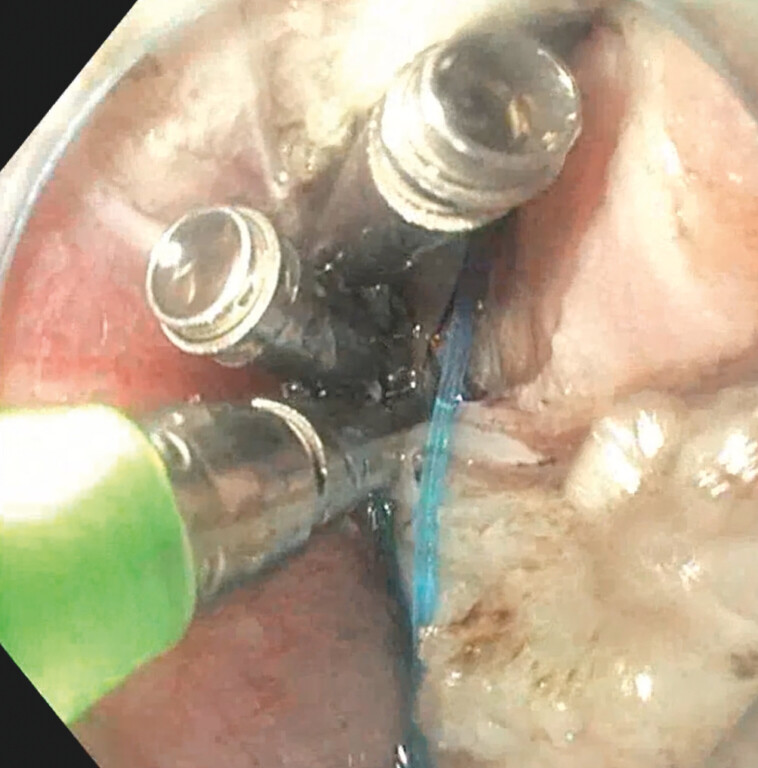
Clips over endoloop at the myotomy site.

**Fig. 8:**
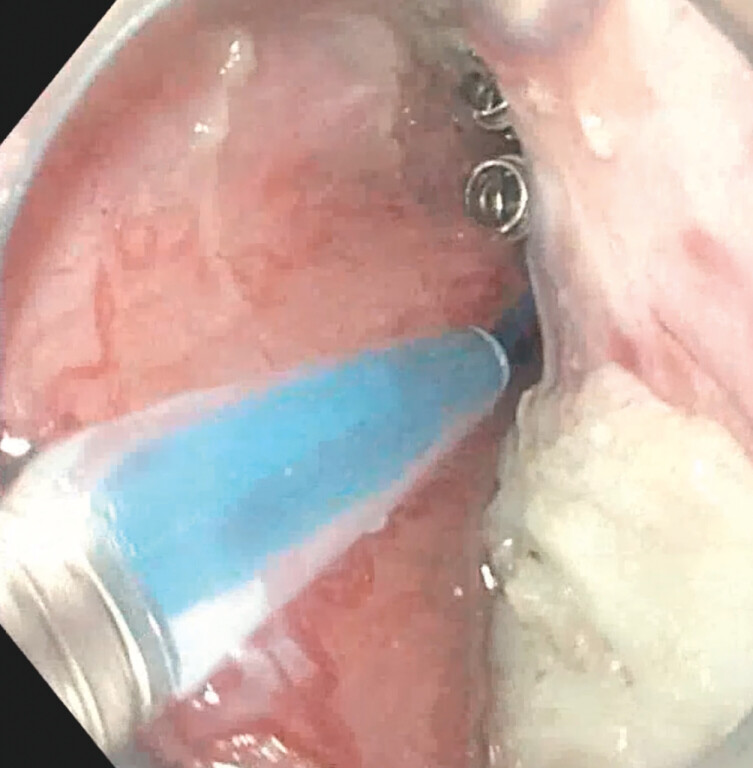
Endoloop tightening.

We performed POEM-F as described previously by Bapaye et. Al 11 . Initially, POEM and POEM-F are performed similarly, with all POEM-F following the standard anterior approach and POEM undergoing either anterior or posterior myotomy. A 2- to 3-cm full-thickness gastric side myotomy is performed. Afterward, an ultraslim endoscope (GIF-H190N, Olympus America, Center Valley, Pennsylvania, United States) is passed into the gastric fundus with a gastroscope (GIF-HQ190; Olympus America) being placed in the submucosal tunnel. The serosa overlying the gastric myotomy is then transected and ( Fig. 1 ) opened and the gastroscope is advanced into the peritoneal cavity ( Fig. 2 ). The gastroscope is then angled upward and leftward to reach the gastric fundus, which is subsequently grasped and retracted into the tunnel to simulate a wrap. When the wrap is deemed satisfactory by visualization from the ultraslim endoscope ( Fig. 3 and Fig. 4 ), the area on the peritoneal side of the stomach is marked using diathermy ( Fig. 5 ) and the gastroscope is removed. An endoloop (HX-400U-30, Olympus) or (Ligation device, LeoMed, PRC) is then grasped with a hemoclip (Lockado, Microtech Corp, PRC) and the gastroscope is reintroduced into the peritoneal cavity. The endoloop is then fixed to the fundus using three endoclips ( Fig. 6 ) and the proximal end of the loop is fixed to the distal myotomy end using three more endoclips ( Fig. 7 ). The endoloop is tightened ( Fig. 8 ) and the proximal end cut by a loop cutter (FS-410, Olympus). Ultimately, the mucosal entry site is closed using more endoclips, after adequate hemostasis is confirmed.

All centers adopted the same protocol for the procedure and all POEM-F cases were performed by the same endoscopist (MK) assisted by a local endoscopist trained in third space endoscopy.

Prior to starting POEM-F in humans, MK performed five procedures successfully in live pigs and subsequently a human case with Bapaye et al. 11 .

### Statistical analysis

Baseline characteristics and results for the study and control cohorts are presented as median (interquartile range) for the continuous variables and total count (percentage) for the categorical variables. The rationale for this decision was that the continuous variables were non-normally distributed and, thus, compared as medians rather than means. Continuous variables between study and control datasets were compared using the Mann-Whitney U test, whereas categorical variables were compared using a chi-square test.

For comparison of the primary and secondary outcomes, a relative risk (RR) was calculated for categorical variables. All statistical analyses were conducted using MedCalc Statistical Software version 23.1.7. All statistical tests were two-sided with a significance level of 0.05 unless otherwise specified.

## Results

### Baseline characteristics

Clinical characteristics of all 64 study participants were divided into POEM-F (n = 31) and control POEM (n = 33) groups, as assessed, and presented in Table 1 . Overall, in the POEM-F cohort, subjects were 48% male with a median age of 52, whereas similarly, the POEM cohort was 58% male with a median age of 58. Pre-POEM GERD symptoms were similar between the POEM-F and the POEM cohorts (41.9% vs 42.4%, *P* = 1). Groups were also similar in the percentage of patients on PPIs pre-procedurally (74% vs 88%) with similar median dosing of 20 mg daily. In addition, median pre-procedural DeMeester score (8 vs 11) and mean percent time with a gastric pH < 4 (2.9% vs 2.6%) was similar in the POEM-F and POEM groups, respectively. Lastly, median pre-POEM Eckardt scores were similar in both groups (7 vs 6).

**Table:** **Table 1** Baseline Characteristics for POEM-F (study) and POEM (control) groups.

Variable	Overall (n = 64)	POEM-F (n = 31)	POEM (n = 33)	*P* value
Age (years)	54.5 (44.5–62.3)	52 (38–63)	58 (49–62)	0.130
Gender (M), n (%)	34 (53.1)	15 (48.4)	19 (57.8)	0.236
BMI (kg/m ^2^ )	22 (19.5–25)	23.5 (20.25–27.5)	21.5 (18.8–24.3)	**0.037**
Eckardt Score (Pre-POEM)	6 (5–7)	7 (5–7.5)	6 (5- 7)	0.360
Gastroesophageal reflux pre-POEM, n (%)	27 (42.2)	13 (41.9)	14 (42.4)	1.000
PPI therapy (pre-POEM), n (%)	52 (79.7)	23 (74.0)	29 (87.9)	0.124
Esophagitis, n (%)	9 (14.1)	3 (9.7)	6 (18.2)	0.474
Barrett’s esophagus, n (%)	2 (3.1)	2 (6.5)	0 (0.0)	0.231
Barrett’s esophagus with dysplasia	0 (0.0)	0 (0.0)	0 (0.0)	
Hiatal hernia, n (%)	8 (12.5)	3 (9.7)	5 (15.2)	0.709
DeMeester score (Pre-POEM), n (%)	9.50 (6.75–12)	8 (4.50–11.50)	11 (8–12)	0.092
Pre-procedure % time pH < 4 (min)	2 (2–3.25)	2 (1–4)	2 (2–3)	0.912
BMI, body-mass index; POEM, peroral endoscopic myotomy; POEM-F, peroral endoscopic myotomy and fundoplication; PPI, proton pump inhibitor.				

### Primary outcomes

Technical success was 100% in both groups. Findings of post-procedure characteristics are reported in Table 2 . POEM-F was always performed anteriorly, whereas in the control group, POEM was performed using either anterior or posterior orientation based on endoscopist preference and 27.3% POEMs in control group were anterior. In the POEM-F group, a composite of cessation or reduction in PPI dosing was achieved in 25 of 31 patients (80.6%) as compared with traditional POEM in eight of 33 patients (24.2%) ( *P* ≤ 0.0001). RR for PPI reduction or cessation in the study vs control group was 3.33 (95% confidence interval [CI] 1.77–6.23, *P* = 0.0002) ( Fig. 1 ). GERD incidence was also lower in patients in the study group, although not significantly (16.1% vs 36.4%, *P* = 0.121) with two patients found to have Los Angeles Classification grade 1 esophagitis in the POEM-F group compared with seven in the POEM group. GERD-HRQL scoring was more also favorable in the study group (6.9 (8.5) vs 28.6 (17.8) ( *P* = 0.001).

**Table:** **Table 2** Procedure characteristics and post-procedure findings.

Variable	Overall (n = 64)	POEM-F (n = 31)	POEM (n = 33)	*P* value
Procedure success	64 (100)	31 (100)	33 (100)	
Anterior vs posterior myotomy (Anterior, %)	40 (62.5)	31 (100)	9 (27.3)	**< 0.0001**
POEM procedure duration (min)	52 (47.75–60.25)	55 (47–66)	52 (48–59)	0.497
Fundoplication duration (min)		17.6 (12–25)	N/A	
PPI cessation (post-POEM), n (%)	23 (35.9)	18 (58.1)	5 (15.2)	**< 0.001**
PPI reduction or cessation, n (%)	33 (51.6)	25 (80.6)	8 (24.2)	**< 0.0001**
Gastroesophageal reflux (post-POEM), n (%)	17 (26.6)	5 (16.1)	12 (36.4)	0.121
Timing of repeat EGD (months post-procedure)	2 (1–2)	1 (1–2.50)	2 (1–2)	0.784
Post-procedure length of stay (days)	1 (1–2)	2 (1–2)	1 (1–2)	0.481
Post-procedure DeMeester score	11 (6–17)	6 (5–11)	16 (12–18)	**< 0.0001**
Post-procedure % time pH < 4	5.50 (2–10)	2 (1–3)	9 (7–11)	**< 0.0001**
Post-procedure Eckardt Score	2 (1–2)	2 (1–2)	2 (1–2)	0.511
Adverse events, n (%) ^*^	7 (11)	3 (9.7)	5 (15.1)	0.24
Need for repeat intervention, n (%) ^†^	4 (6.3)	0 (0)	4 (12.1)	
Eckardt score at last follow-up	1.50 (1–2)	1 (1–2)	2 (1–2)	0.053
Longest follow-up time (months)	9 (7–12)	8 (6–10.50)	11 (9–13)	**0.37**
EGD, esophagogastroduodenoscopy; POEM, peroral endoscopic myotomy; POEM-F, peroral endoscopic myotomy and fundoplication; PPI, proton pump inhibitor. ^*^ Adverse events: There were three mucosal injuries (9.7%) in the POEM-F group and four (12.1%) in the POEM group. One patient had post-procedure aspiration pneumonia in the POEM group. **^†^** Repeat procedure in the POEM group was Dor fundoplication in all four patients.				

**Fig. 9:**
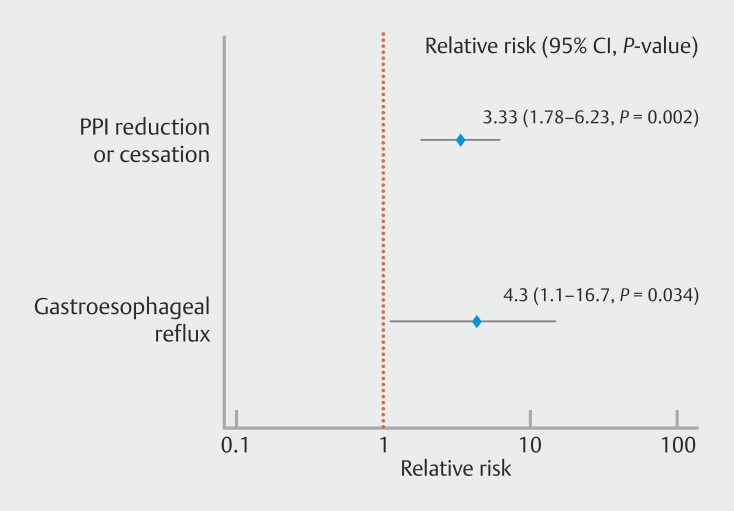
Forest plot of relative risk (RR) of PPI reduction and GERD symptoms after POEM-F compared with traditional POEM.

However, RR of GERD absence in the study vs control groups was significant, RR = 4.3 (95% CI 1.1–16.7, *P* = 0.034) ( Fig. 9 ). Mean along with median percent time with pH < 4 was also significantly lower in the POEM-F group (2.75 ± 2.53 vs 9.3 +/- 3.6, *P* ≤ 0.0001). Post-procedure DeMeester scores were also lower in POEM-F (median = 6) than POEM (median = 16) recipients ( *P* ≤ 0.0001), with a majority under the cutoff value of 14.7 in the POEM-F group (27/31, 87%) vs POEM group with a minority under the cutoff (12/33, 36.3%). Median Eckardt scores were significantly lower post-procedurally; however, they were similar between both cohorts. Mean and median Eckardt scores were also maintained at time of last follow-up.

### Secondary outcomes

Secondary outcomes are reported in Table 2 . Procedures for both POEM-F and traditional POEM were equally well tolerated with minimal AEs. There was no statistical difference in procedure length between the two groups in performing POEM; however, fundoplication adds an extra 17.6 minutes on average with a range between 12 to 25 minutes in the POEM-F group. There were three mucosal injuries (9.7%) in the POEM-F group and four (12.1%) in the POEM group ( *P* = 0.24). One patient had post-procedure aspiration pneumonia in the POEM group. Between the study and control groups, post-procedure length of hospital stay (1.6 vs 1.5 days) and procedure time (55 min vs 52 mins) were not statistically significantly different. In the POEM cohort, 12% of patients required repeat interventions (4 patients—Dor fundoplication) 2 patients underwent and anterior approach whereas the other two had a posterior approach, although no repeat interventions were indicated in the POEM-F group at the 6-month follow-up.

## Discussion

Our study compared outcomes of POEM-F to traditional POEM, focusing on reflux control and reduction in medication usage, achalasia symptomatic relief, and procedure safety. The findings suggest that POEM-F offers superior reflux management while maintaining similar efficacy in symptom relief and procedure safety compared with traditional POEM.

Traditional POEM was first performed in 2008 in Japan 3 . The same group similarly piloted the POEM-F procedure subsequently to prevent post-POEM GERD in 2019 12 . There have been refinements in the procedural aspects of the fundoplication technique, especially in endoscopic techniques for the fundoplication portion. In many studies from Japan, the method for endoscopic hand suturing (EHS) in fundoplication, originally for gastric mucosal defects, was adapted from Goto et. Al, which has been used instead of endoclips to prevent remaining foreign bodies in situ 13 . This is done via three steps with endoscopic stitches, as described by Toshimori et. Al in 2020 14 . First, a submucosal tunnel is created in the anterior wall of the lower esophagus using a gastroscope with a triangle-tipped knife. Then, fundoplication is achieved by advancing a submucosal endoscope through the peritoneum into the abdominal cavity. With a surgical suture needle held by an endoscopic needle holder, a distal anchoring stitch is made at the anterior gastric wall by grasping the full gastric wall. A second stitch is placed distally at the end of the dissected muscle in the submucosal tunnel, and the suture is tightened, partially wrapping the gastric cardia. The final stitch is thrown to reinforce the fundoplication and then cut with endoscopic scissors. Mucosal closure is then achieved by clipping the entry site, as in the traditional POEM procedure. There have not been comparative studies regarding EHS versus endoclip for endoscopic fundoplication outcomes; however, prior case control studies have described mucosal closure with EHS having higher operative time and higher total closure costs when compared with endoclips 15 . Further, we hypothesize that scar tissue formation surrounding the endoclip provides greater long-term stability to prevent regression of the fundoplication when compared with EHS, although further studies are required to demonstrate POEM-F outcomes.

In the United States, the first POEM-F procedure was performed in 2022 and the first case series was described in 2023 by Shrigiriwar using endoclips for the fundoplication portion 16 . Overall feasibility and safety of this procedure have been further studied and validated in multiple studies to date 17 .

To the best of our knowledge, this is the largest study comparing outcomes of patients undergoing either POEM-F or POEM procedures. Both study cohorts were well-matched in terms of demographic and clinical characteristics, ensuring a balanced comparison. Although incidence of pre-POEM reflux symptoms was lower in the POEM-F group, this difference was not statistically significant (22.5% vs. 42%, *P* = 0.091). Other baseline characteristics, including the proportion of patients on PPI therapy, pre-procedure DeMeester scores, and Eckardt scores, were also similar between the groups. A caution point to remember is that in achalasia, a substantial proportion of these patients have a false-positive pH study due to fermentation-related acid production.

Procedurally, our POEM-F procedure was performed as described by Shrigiriwar et. Al. and does not employ the EHS technique as previously noted above by Toshimori et. Al 14 .

Although technical success was achieved in both groups, significant differences were observed in post-procedure reflux control. The composite outcome of cessation or reduction in PPI use was notably higher in the POEM-F group (80%) compared with the POEM group (24%) ( *P* ≤ 0.00001). In addition, mean percent time with esophageal pH < 4 was significantly lower in the POEM-F group, as were median post-procedure DeMeester scores. As expected, these findings indicate that POEM-F is superior in mitigating post-procedure reflux, a known complication of traditional POEM. Importantly, Eckardt scores, a measure of symptom relief in achalasia, improved significantly in both groups, with no significant difference post-procedure or at the time of last follow-up 8 to 10 months post-procedurally. The addition of fundoplication to POEM did not compromise the primary outcome of symptomatic achalasia relief.

Both procedures were also well tolerated with minimal AEs. Rates of mucosal injury were comparable (9.7% in POEM-F vs. 12.1% in POEM, *P* = 0.24) and one case of post-procedure aspiration pneumonia was observed in the POEM group. Length of hospital stay and procedure time did not significantly differ between groups, further supporting the safety profile of POEM-F with a negligible increase in procedure time when adding endoscopic fundoplication. Notably, four patients in the POEM cohort required subsequent GERD intervention. In all four cases, this was a surgical Dor fundoplication, reinforcing the benefit of incorporating endoscopic fundoplication in preventing GERD symptoms and future surgical intervention in patients.

Despite the strengths of our study, limitations exist. The minimum duration of 6 months may not fully capture long-term outcomes, particularly regarding reflux control and durability of symptom relief. In addition, there are many reported post-endoscopic fundoplication complications and recurrence of symptoms after 12 months, as well as the need for reintroduction of PPI therapy 18 . Lastly, the POEM-F procedure is complex and requires two trained advanced endoscopists to perform it. On the other hand, the multicenter nature of our study demonstrates that the procedure is reproducible across various centers with proper training and expertise, but consideration must be given to the additional time required (up to 25 min in difficult cases) and the need to familiarize the team with other accessories and techniques which might have an educational and economic impact.

## Conclusions

In conclusion, POEM-F effectively addresses the major limitation of traditional POEM—post-procedure GERD—without compromising achalasia-related symptom relief or procedure safety. Marked reduction in acid exposure and reliance on PPI therapy underscores the advantage of POEM-F in reflux control. Moreover, lack of need for additional anti-reflux interventions in POEM-F patients suggests that this technique may reduce the burden of GERD. However, further studies, including randomized controlled trials with longer-term follow-up, are needed to validate these findings.

## Notes

Correction **Safety and efficacy of peroral endoscopic myotomy with endoscopic fundoplication compared with POEM alone: International multicenter cohort study** Michel Kahaleh, Vera Hapshy, Juan A. Alcívar et al. Endoscopy International Open 2025; 13: a26556550. DOI: 10.1055/a-2655-6550 In the above-mentioned article an author's affiliation was corrected. This was corrected in the online version on 20.08.2025.
