# Correction: Safety and efficacy of peroral endoscopic myotomy with endoscopic fundoplication compared with POEM alone: International multicenter cohort study

**DOI:** 10.1055/a-2685-7125

**Published:** 2025-08-19

**Authors:** Michel Kahaleh, Vera Hapshy, Juan A Alcívar, Jorge Baquerizo-Burgos, Hannah Lukashok, Monica R Gaidhane, Iman Andalib, Amy Tyberg, Avik Sarkar, Haroon Shahid, Abid Allehibi, Resheed Alkhiari, Magda L Rodriguez, Carmen Bautista-Altamirano, Sarbelio Rodriguez, Maria G Porfilio, Mine Carames, Juan Carlos Carames, Amol Bapaye, Carlos Robles-Medranda

**Affiliations:** 1 Foundation of Interventional and Therapeutic Endoscopy, New York, United States; 2 23498 Gastroenterology, Hackensack Meridian Jersey Shore University Medical Center, Neptune City, United States; 3 Instituto Ecuatoriano de Enfermedades Digestivas, Guayaquil, Ecuador; 4 24263 Gastroenterology, Hackensack Meridian JFK University Medical Center, Edison, United States; 5 23498 Medicine, Hackensack Meridian Jersey Shore University Medical Center, Neptune City, United States; 6 37849 Gastroenterology, King Fahad Medical City, Riyadh, Saudi Arabia; 7 37850 Gastroenterology, King Saud University, Riyadh, Saudi Arabia; 8 537694 Gastroenterology, Our Lady of Remedies Clinic, Cali, Colombia; 9 Bariatric Endoscopy Unit, Hospital Universitario Ruber Juan Bravo, Madrid, Spain; 10 Endoscopy Unit, Service of Gastroenterology, Hospital 12 de Octubre, Madrid, Spain; 11 Gastroenterology, Escuela Dr.Ramon Madariaga, Posadas, Argentina; 12 Gastroenterology, Santander Hospital, Reynosa, Mexico; 13 Shivanand Desai Center for Digestive Disorders, Deenanath Mangeshkar Hospital and Research Center, Pune, India; 14 Endoscopy, Omni Hospital, Guayaquil, Ecuador



In the above-mentioned article an author's affiliation was corrected. This was corrected in the online version on 20.08.2025

